# Induction of apoptosis and suppression of tumor growth by Nur77-derived Bcl-2 converting peptide in chemoresistant lung cancer cells

**DOI:** 10.18632/oncotarget.25437

**Published:** 2018-05-25

**Authors:** Martin C. Pearce, John T. Gamble, Prasad R. Kopparapu, Edmond F. O'Donnell, Monica J. Mueller, Hyo Sang Jang, Julie A. Greenwood, Arnold C. Satterthwait, Robert L. Tanguay, Xiao-Kun Zhang, Siva Kumar Kolluri

**Affiliations:** ^1^ Cancer Research Laboratory, Department of Environmental & Molecular Toxicology, Oregon State University, Corvallis, Oregon 97331, USA; ^2^ Department of Biochemistry & Biophysics, Oregon State University, Corvallis, Oregon 97331, USA; ^3^ Sanford Burnham Prebys Medical Discovery Institute, La Jolla, CA 92031, USA; ^4^ Department of Environmental & Molecular Toxicology, Environmental Health Sciences Center, Oregon State University, Corvallis, Oregon 97331, USA; ^5^ Linus Pauling Institute, Oregon State University, Corvallis, OR 97331, USA; ^6^ Center for Genome Research and Biocomputing, Oregon State University, Corvallis, OR 97331, USA

**Keywords:** chemoresistance, Bcl-2, NuBCP-9, paclitaxel, lung cancer

## Abstract

Resistance to chemotherapy is a major cause of treatment failure and poor overall survival in patients with lung cancer. Identification of molecular targets present in resistant cancer cells is essential for addressing therapeutic resistance and prolonging lung cancer patient survival. Members of the B-cell lymphoma 2 (Bcl-2) family of proteins are associated with chemotherapeutic resistance. In this study, we found that pro-survival protein Bcl-2 is upregulated in paclitaxel resistant cells, potentially contributing to chemotherapy resistance. To exploit the increase in Bcl-2 expression for targeting therapy resistance, we investigated the effects of a peptide derived from the nuclear receptor Nur77 that converts Bcl-2 from an anti-apoptotic protein to a pro-apoptotic protein. The Nur77 derived peptide preferentially induced apoptosis in paclitaxel-resistant cancer cells with high expression of Bcl-2. This peptide also induced apoptosis of multidrug resistant H69AR lung cancer cells that express Bcl-2 and inhibited their growth in 3D spheroids. The Nur77 peptide strongly suppressed the growth of paclitaxel-resistant lung cancer cells in a zebrafish xenograft tumor model. Taken together, our data supports a new strategy for treating lung cancers that acquire resistance to chemotherapy through overexpression of Bcl-2.

## INTRODUCTION

Lung cancer is the second most common cancer in men and women, and is the leading cause of mortality from cancer [1]. The standard of care for lung cancer comprises of surgical resection, chemotherapy and radiation therapy [2]. Chemotherapeutic options are critical for the treatment of advanced lung cancer. Taxanes such as paclitaxel are antimitotic agents currently used as standard of care first line therapy either alone, or in combination with platinum-based agents for both primary and advanced lung cancer [3, 4]. Paclitaxel interferes with tubulin to stabilize microtubule composition and normal spindle assembly resulting in inhibition of mitosis [5]. Paclitaxel is also used as a single agent in advanced and metastatic lung cancer where surgery is not an option [4, 6]. However, acquired resistance to paclitaxel is common, which is associated with poor prognosis, and limited therapeutic options once resistance occurs [7, 8]. Doxorubicin is another chemotherapeutic which is used for treatment of recurrent small cell lung cancer (SCLC) and resistance to doxorubicin has also been reported [9, 10]. Chemotherapy is the standard treatment for SCLC and while initial response to chemotherapy is good, most SCLC patients develop multidrug resistance [11]. Cross-resistance to other chemotherapy drugs after treatment with a single agent reduces treatment options, contributing significantly to the 5-year patient survival of less than 5% [12]. For example, overexpression of multidrug resistance associated protein (MRP1) results in export of anticancer drugs [13]. Therefore, it is critical to identify molecular targets and novel approaches to selectively induce death in paclitaxel resistant cancer cells.

The Bcl-2 family of proteins are key regulators of cell death, and include both anti-apoptotic and pro-apoptotic members [14, 15]. Each protein of this family possesses at least one of four conserved motifs called Bcl-2 homology (BH) domains. The anti-apoptotic proteins include Bcl-2, Bcl-xL and Mcl-1, which possess BH1-4 domains. On the other hand, the pro-apoptotic Bcl-2 family members are divided into two subclasses, comprising the BH3 only proteins such as Noxa and Puma and multi-domain proteins such as Bak and Bax which possess the BH1-3 domains [14]. The BH3 domain is considered as the death domain [15]. Many anticancer agents such as doxorubicin induce tumor killing through activation of the Bcl-2 family member regulated apoptotic pathway [14, 16, 17]. In addition, expression of BH3-only proteins is induced by certain anticancer agents such as paclitaxel, resulting in activation of Bak/Bax and induction of apoptosis [18]. Elevated expression of anti-apoptotic proteins such as Bcl-2 can prevent apoptosis induction through preventing Bak/Bax activation [19]. Upregulation of Bcl-2, and other anti-apoptotic proteins, is a potential mechanism of acquired resistance to many therapeutic agents [20–22].

The ability to target and induce death of cancer cells with high levels of Bcl-2 represents a unique opportunity to treat chemoresistant cancers. Current strategies of targeting Bcl-2 include peptides derived from the BH3 domain of pro-apoptotic Bcl-2 family members and small molecule inhibitors that neutralize the pro-survival function of Bcl-2. The Bcl-2 inhibitor ABT-199 (Venetoclax®) has been approved for treatment of chronic lymphocytic leukemia (CLL) [23, 24]. ABT-199 binds to the BH3 binding pocket of Bcl-2, displacing the endogenous BH3-only proteins, which in turn induces cell death via a Bax/Bak dependent mechanism [25].

Nur77 (also known as NGFI-B and TR3) is an immediate-early and an orphan member of the steroid/thyroid/retinoid receptor superfamily that act as transcriptional factors to regulate gene expression [26]. Nur77 plays a critical role in cancer cell survival and proliferation [27]. Subcellular localization of Nur77 dictates its biological function and growth or death of various cancer cells [26, 28]. Nur77 migrates from the nucleus to mitochondria to initiate apoptosis. Nur77 interacts with Bcl-2, resulting in mitochondrial localization of Nur77, induction of cytochrome *c* release and apoptosis [28, 29]. During the course of identifying the minimal functional domain of Nur77, we discovered a nine amino acid peptide, NuBCP-9 that mimics the mechanistic and functional activities of Nur77 [28, 30]. Thus, Bcl-2 can be targeted by Nur77 derived peptides that convert Bcl-2 from an anti-apoptotic to a pro-apoptotic protein [26, 30, 31]. NuBCP-9 binds to the Bcl-2 loop domain and induces a conformational change in the protein, exposing the Bcl-2 BH3 domain, and ultimately converting Bcl-2 into a pro-apoptotic state [28, 30]. This provides an opportunity to overcome mechanisms of drug resistance, as NuBCP-9 effects are potentiated in cells with high expression of Bcl-2 [15, 30].

In the current study, we derived paclitaxel resistant H460 non-small cell lung cancer cells and identified an increase in Bcl-2 expression as well as cross resistance to doxorubicin. Multidrug resistant lung cancer H69AR cells derived from H69 also have high expression of Bcl-2 [11]. NuBCP-9 preferentially induced apoptosis in the paclitaxel resistant H460 and the multidrug resistant lung cancer cells. NuBCP-9 strongly suppressed growth of paclitaxel resistant lung cancer cells in a zebrafish xenograft model. These results provide a new strategy of targeting and eliminating chemotherapy resistant cancer cells through Bcl-2 functional conversion.

## RESULTS

**Figure 1:**
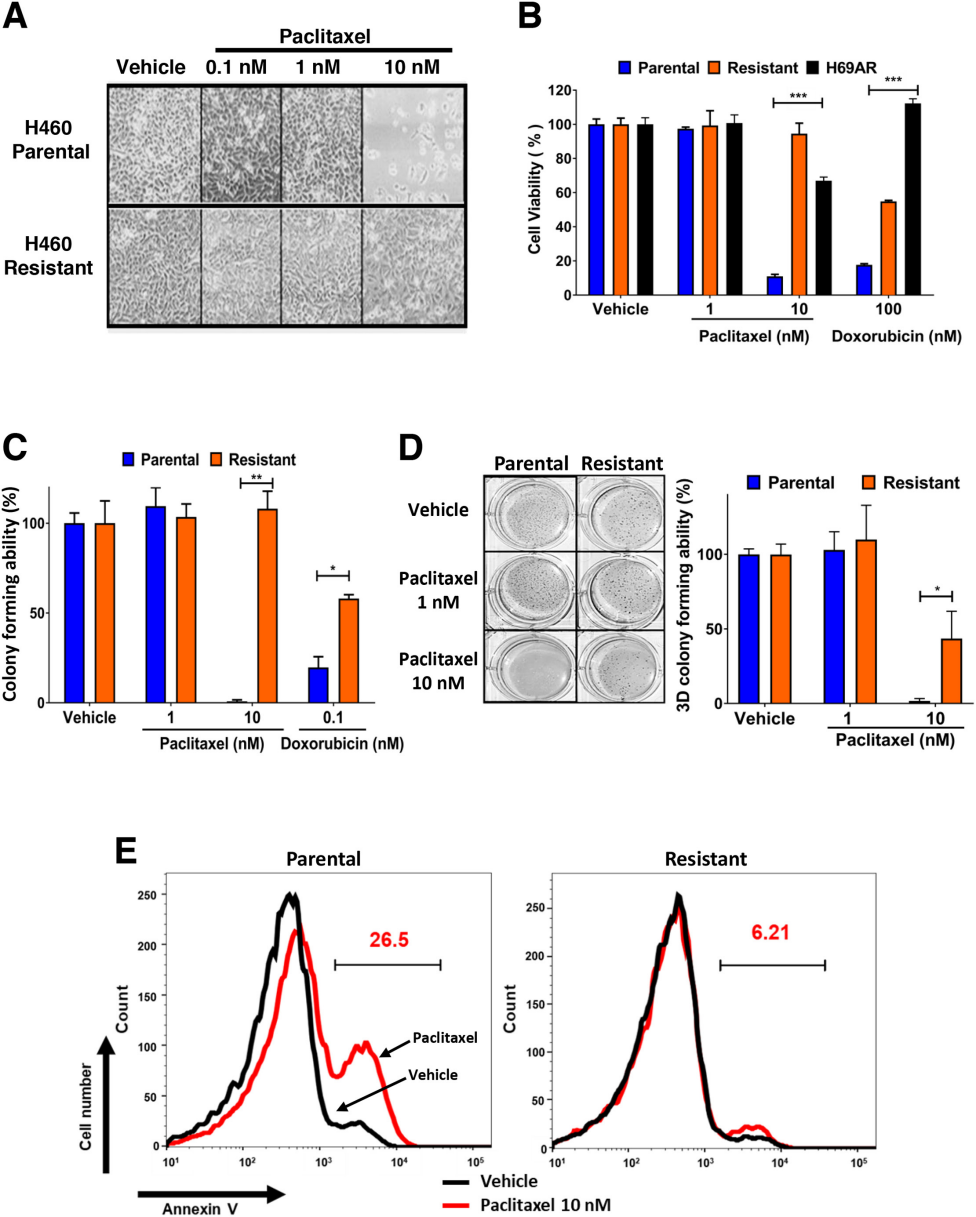
Establishment of paclitaxel resistant H460 lung cancer cells and their cross resistance to doxorubicin **(A)** H460 parental and paclitaxel resistant lung cancer cells were plated and treated with indicated concentrations of paclitaxel, images were captured at 10x magnification after 48 hours. **(B)** Effect of paclitaxel, doxorubicin on H69AR multidrug resistant and H460 parental and derived paclitaxel resistant lung cancer cells after 72 hours of treatment. Percentage viability is calculated relative to vehicle treatment. Data is representative of three independent assays done in triplicate. One-way ANOVA with Dunnett's multiple comparisons post-test, ^***^P<0.0001. **(C)** Clonogenic survival assays with H460 parental and resistant cells treated continuously for 14 days with vehicle or indicated concentration of paclitaxel and doxorubicin. Colony forming ability (%) is calculated from the number of colonies relative to vehicle treatment. Data is representative of three independent assays conducted in triplicate. Two-way ANOVA with Dunnett's multiple comparisons post-test, ^*^P<0.05, ^**^P<0.001. **(D)** 3D soft agar tumorigenicity assay with H460 parental and resistant cells treated continuously for 14 days with vehicle or indicated concentration of paclitaxel (colonies indicated in blue). 3D colony forming ability (%) is calculated relative to vehicle treatment. Two-way ANOVA with Dunnett's multiple comparisons post-test, ^*^P<0.05. **(E)** Annexin V staining of H460 cells treated for 48 hours with vehicle or paclitaxel 10 nM. Histogram gate indicates percentage of apoptotic cells after paclitaxel treatment. Black line, Vehicle; Red line, Paclitaxel 10 nM. Results are the representative of three independent experiments.

We derived paclitaxel resistant cancer cells to ascertain if Bcl-2 expression is altered during the development of chemoresistance and to determine if Bcl-2 functional converting peptides can be used to selectively kill paclitaxel resistant lung cancer cells. H460 lung cancer cells are extremely sensitive to 10 nM paclitaxel and 100 nM doxorubicin (Figure 1A-1C). H460 cells were treated with paclitaxel over a period of 6 weeks to derive paclitaxel resistant cells (Figure 1A-1C). The derived paclitaxel resistant H460 cells had similar level of resistance to paclitaxel as the multidrug resistant H69AR lung cells (Figure 1B) [32]. Paclitaxel inhibited the ability of parental cells to form colonies in 3D soft agar assays, while paclitaxel resistant H460 cells were unaffected (Figure 1D). The H460 paclitaxel resistant cells were also less responsive to doxorubicin treatment, indicating cross chemoresistance (Figure 1B-1C). There was minimal induction of apoptosis in paclitaxel resistant H460 cells compared to parental H460 cells after exposure to 10 nM paclitaxel for 48 hours (Figure 1E).

### Anti-apoptotic Bcl-2 is upregulated in paclitaxel resistant lung cancer cells

**Figure 2:**
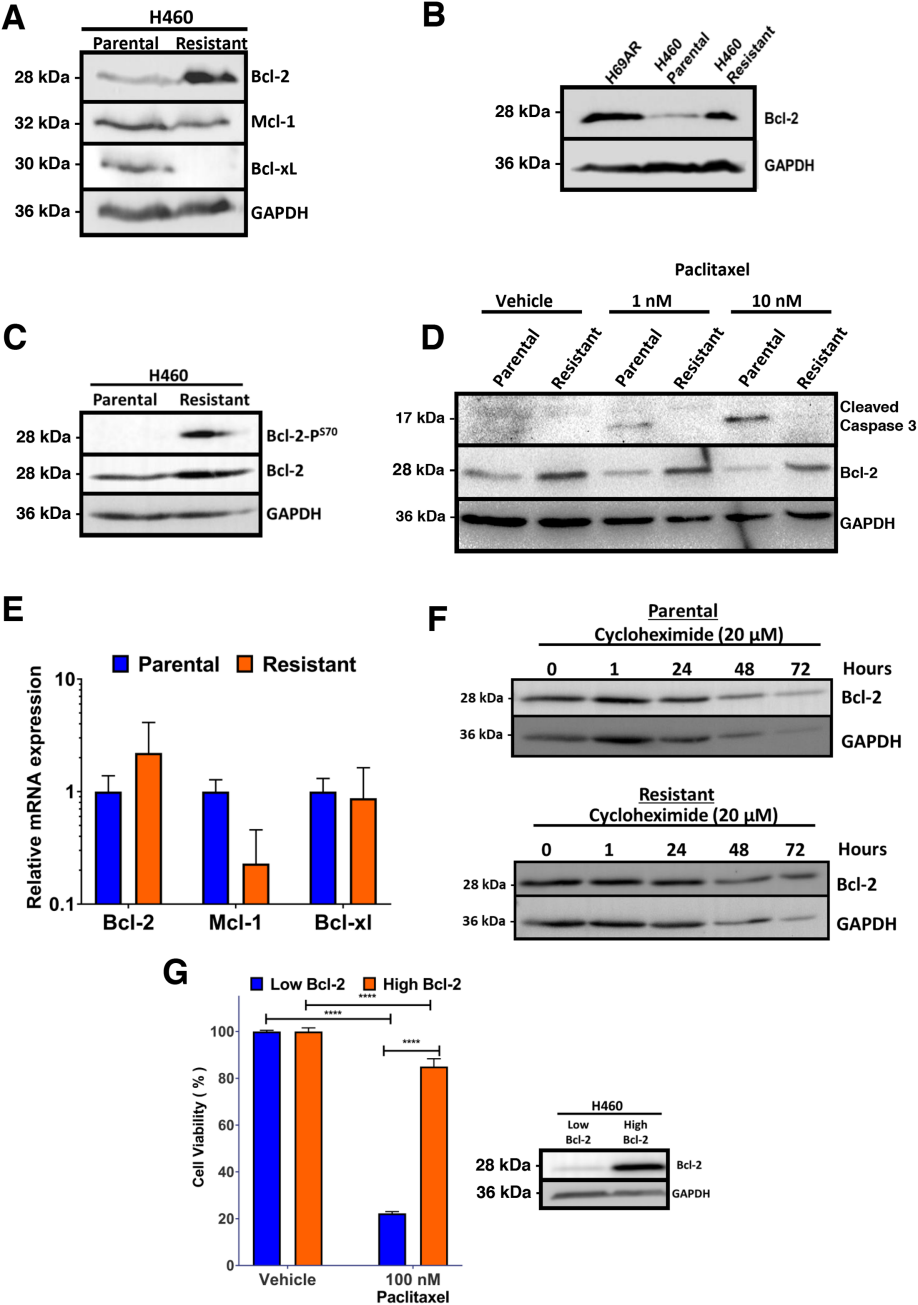
Paclitaxel resistant H460 cells express elevated levels of Bcl-2 **(A)** Increased Bcl-2 expression in Paclitaxel resistant H460 cells. Western blot analysis of H460 parental and derived paclitaxel resistant cells. **(B)** High Bcl-2 expression in multidrug resistant H69AR and paclitaxel resistant H460 cells compared to parental H460 cells. Western blot analysis of multidrug resistant H69AR and H460 parental and paclitaxel resistant cells probed with indicated antibodies. **(C)** Increased phosphorylation of Bcl-2 at serine 70 in H460 paclitaxel resistant cells. Western blot analysis of H460 parental and paclitaxel resistant cells blotted with indicated antibodies. **(D)** Correlation of high Bcl-2 and increased resistance to paclitaxel. Western blot analysis of H460 parental and paclitaxel resistant cells treated with paclitaxel for 48 hours and probed with indicated antibodies. **(E)** Analysis of Bcl-2, Mcl-1 and Bcl-xL mRNA levels in parental and resistant H460 cells using quantitative real-time PCR. **(F)** Bcl-2 protein stability is not altered in parental and paclitaxel resistant cells. The level of Bcl-2 was detected by Western blot and GAPDH was used as a loading control. **(G)** Bcl-2 expression alone confers resistance to paclitaxel. Flow cytometry-based analysis of viability in H460 parental cells expressing Bcl-2 or control vector, after treatment with 100 nM paclitaxel for 48 hours; right panel: Western blot analysis of transfected cells show increased Bcl-2 expression. Results are the mean±s.d. Two-way ANOVA, with Sidaks multiple comparison post-test, ^****^ = P<0.0001.

Changes in expression of Bcl-2 family members is a potential mechanism of resistance [33]. Assessment of the levels of Bcl-2 family of proteins identified an increase in Bcl-2 expression in the paclitaxel resistant cell line (Figure 2A). A reduction in Bcl-xL expression was observed, whereas Mcl-1 levels were unchanged (Figure 2A). Interestingly, H69AR multidrug resistant cells also express high levels of Bcl-2 (Figure 2B). Paclitaxel resistant H460 cells were determined to have phosphorylation of Bcl-2 at serine 70 within the unstructured loop, which has been linked with its increased anti-apoptotic function (Figure 2C) [34, 35]. Paclitaxel induced cleavage of caspase 3 in parental H460 cells indicating apoptosis, but not in resistant cells confirming resistance to paclitaxel (Figure 2D). We determined if the increased expression of Bcl-2 in paclitaxel resistant H460 cells was a result of increased mRNA expression. There was no statistically significant change in Bcl-2 mRNA expression, whereas Mcl-1 mRNA levels were reduced in paclitaxel-resistant H460 (Figure 2E). To determine if increased Bcl-2 in paclitaxel resistant cells was due to increased protein stability, protein synthesis inhibitor, cycloheximide treatment was used to block new protein synthesis and levels of Bcl-2 was determined by Western blot. There was no difference in Bcl-2 protein stability between parental and paclitaxel resistant H460 cells (Figure 2F). To determine if expression of Bcl-2 alone confers resistance to paclitaxel, parental H460 cells were transfected with Bcl-2 expression vector (Figure 2G). The Bcl-2 high expressing H460 cells were unresponsive to paclitaxel, indicating Bcl-2 expression alone confers resistance (Figure 2G).

### Expression of Nur77 peptide induces apoptosis preferentially in paclitaxel resistant lung cancer cells

**Figure 3:**
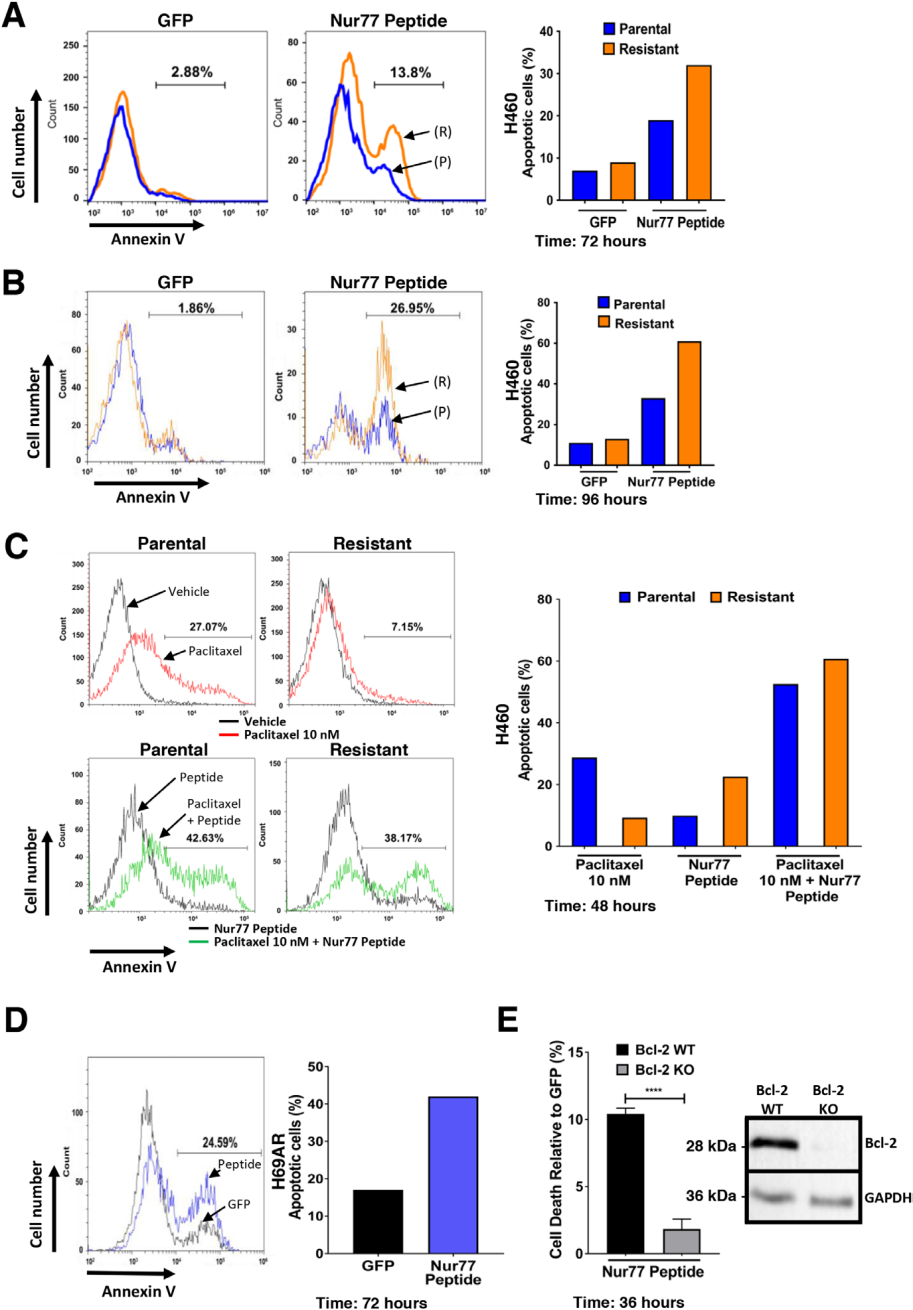
Expression of Nur77 derived peptide in H460 cells induces apoptosis preferentially in paclitaxel resistant H460 lung cancer cells **(A)** Annexin V staining of H460 parental and paclitaxel resistant cells after expression of control GFP or GFP-Nur77 peptide for 72 hours. Histogram gate indicates difference in annexin V positive cells between H460 parental (P) (blue) and paclitaxel resistant (R) (orange) cells. Data is representative of 3 independent experiments and quantification of apoptosis is shown in the right panel. **(B)** Annexin V staining of H460 parental (P) (blue) and resistant (R) (orange) cells after expression of control GFP or GFP-Nur77 peptide for after 96 hours. Histogram gate indicates difference in annexin V positive cells between H460 parental and paclitaxel resistant cells. Data is representative of 3 independent experiments and quantification of apoptosis is shown in the right panel. **(C)** Annexin V staining of H460 parental and resistant cells after expression of GFP-Nur77 peptide with or without co-treatment with paclitaxel (10 nM). Histogram gates indicate difference in annexin V positive cells between vehicle and paclitaxel 10 nM (Upper panels), Nur77 peptide expression alone and Nur77 expression combined with paclitaxel 10 nM (Lower panels). Data is representative of 3 independent experiments and quantification of apoptosis is shown in the right panel. **(D)** Annexin V staining of H69AR multidrug resistant lung cancer cells after expression of control GFP (black) or GFP-Nur77 peptide (blue), histogram gate indicates difference in annexin V positive cells between GFP and GFP-Nur77 peptide expression. The right panel is the quantification of percentage of apoptotic cells from the histograms. **(E)** Expression of Nur77 peptide induces cell death in a Bcl-2-dependent manner in multidrug resistant H69AR lung cancer cells. Flow cytometry-based analysis of cell death in Bcl-2 knockout H69AR CRISPR lines after expression of GFP-Nur77 peptide for 36 hours relative to control GFP expression. Suppression of Bcl-2 expression is confirmed by Western blot analysis of H69AR CRISPR lines expressing control CRISPR plasmid (Bcl-2 WT) and Bcl-2 targeted gRNA CRISPR plasmid (Bcl-2 KO). Results are the of mean±s.d. unpaired two-tailed t-test ^****^=P<0.0001.

We previously reported that nuclear receptor Nur77 migrates to mitochondria to induce apoptosis by converting Bcl-2 from an anti-apoptotic protein to a pro-apoptotic protein [28, 30, 36]. We derived peptides from Nur77 Bcl-2 binding region that induced apoptosis in a Bcl-2 dependent manner [30]. We next determined if the increased expression of Bcl-2 in paclitaxel resistant H460 cells could be targeted by Nur77 peptides. Control green fluorescent protein (GFP) expression had no effect on parental or resistant H460 cells. In contrast, Nur77 peptide expression induced apoptosis in 18% of parental cells and 31% of paclitaxel resistant H460 cells (Figure 3A). The difference became more apparent with time, with greater than 60% of paclitaxel-resistant H460 cells staining positive for annexin V after 96 hours compared to 30% of the parental cells (Figure 3B). Taken together, these data indicate that expression of Nur77 peptide induces cell death preferentially in paclitaxel resistant H460 cancer cells. Furthermore, Nur77 peptide expression in combination with paclitaxel treatment was more effective than peptide expression alone as the combination induced apoptosis in approximately 60% of both parental and paclitaxel resistant H460 cells (Figure 3C). Thus, paclitaxel together with Nur77 peptide expression increased apoptosis by 40% in both parental and resistant cells after 48 hours (Figure 3C). Expression of Nur77 peptide also induced apoptosis (43%) in multidrug resistant H69AR cells (Figure 3D). To determine the requirement of Bcl-2 for Nur77 peptide-induced effects in the multidrug resistant H69AR cells, Bcl-2 knock out cells were derived using CRISPR-Cas9. Suppression of Bcl-2 expression was confirmed by Western blot (Figure 3E). Expression of Nur77 peptide resulted in apoptosis of Bcl-2 expressing cells, but not in Bcl-2 non-expressing H69AR indicating the requirement of Bcl-2 (Figure 3E).

### NuBCP-9 treatment induces apoptosis in paclitaxel resistant lung cancer cells

**Figure 4:**
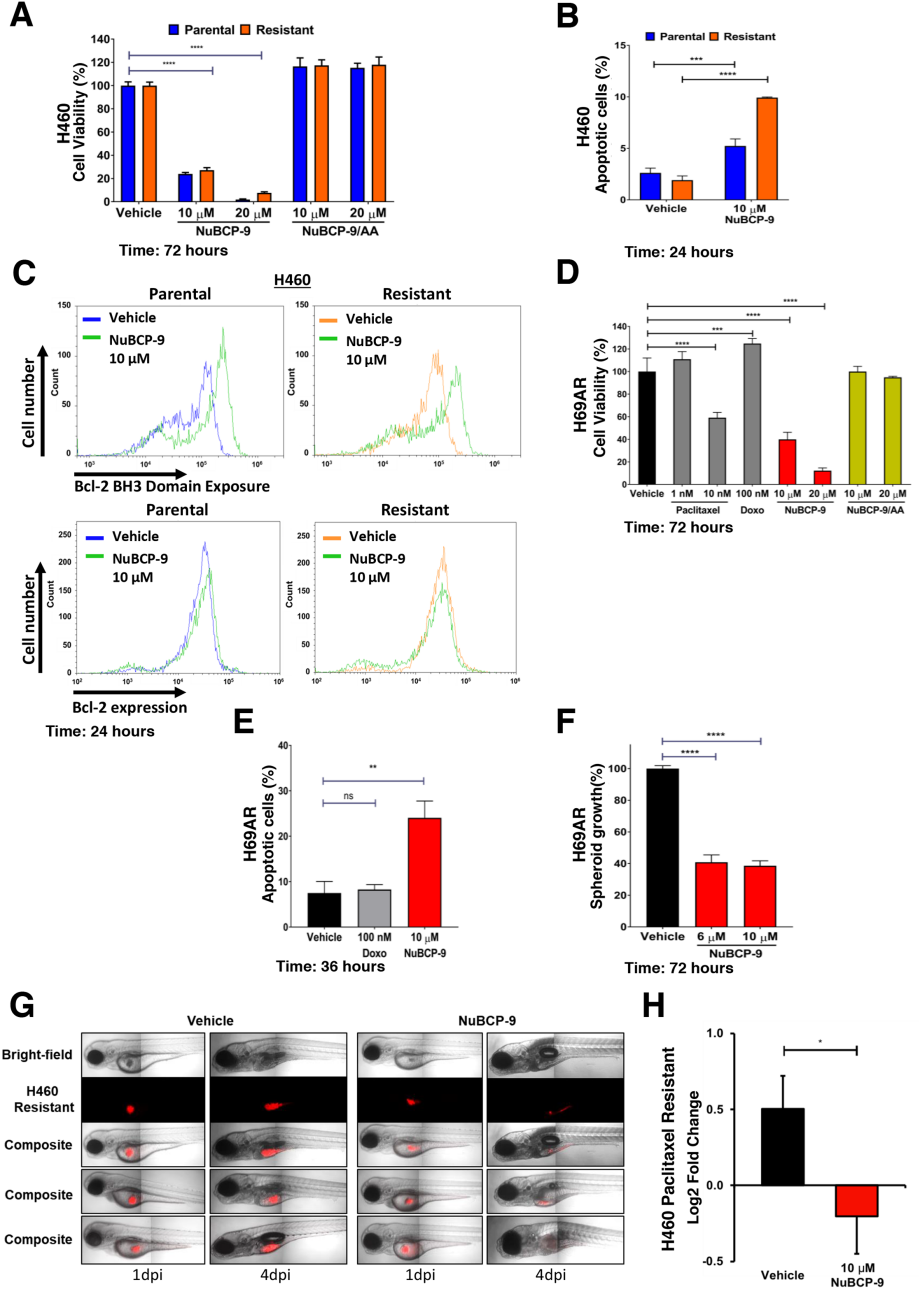
NuBCP-9 suppresses H460 paclitaxel resistant lung cancer cell growth in a xenograft zebrafish model **(A)** Effect of NuBCP-9, NuBCP-9/AA (inactive form) on H460 parental and paclitaxel resistant lung cancer cells after 72 hours of treatment in 1% serum. Two-way ANOVA with Dunnett's multi comparisons post-test, ^****^=P<0.0001. **(B)** Annexin V staining of H460 parental and paclitaxel resistant cells treated with vehicle or 10 μM NuBCP-9 for 24 hours in 1% serum. Results are the mean±s.d. of three technical replicates. Two-way ANOVA with Dunnett's multi comparisons post-test, ^***^=P<0.001, ^****^=P<0.0001. **(C)** NuBCP-9 induces conformational change in Bcl-2 and exposes its BH3 domain. Parental and Resistant H460 cells were treated for 24 hours with vehicle or NuBCP-9 (10 μM) and immunostained with Bcl-2 BH3 specific antibody (upper panel), or Bcl-2 conformation independent antibody (lower panel) and analyzed by flow cytometry. Shift of peak to the right indicates BH3 domain exposure in upper panel. There is no such shift in the lower panels. **(D)** Effect of Paclitaxel, Doxorubicin (Doxo), NuBCP-9 and NuBCP-9/AA (inactive form) on multidrug resistant H69AR lung cancer cells after 72 hours of treatment in 1% serum. Two-way ANOVA with Dunnett's multi comparisons post-test, ^***^=P<0.001, ^****^=P<0.0001. **(E)** Annexin V staining of H69AR multidrug resistant cancer cells treated with vehicle, doxorubicin (100 nM) or NuBCP-9 (10 μM) for 36 hours. Results are the mean±s.d. of three technical replicates. Unpaired student t-test, ^**^=P<0.01. **(F)** NuBCP-9 reduces growth of multidrug resistant lung cancer cell 3D spheroids. H69AR spheroid cultures were grown for 48 hours and were then treated for 72 hours with vehicle, NuBCP-9 (6 and 10 μM). Percentage viability is calculated relative to vehicle treatment. Unpaired student t-test, ^****^=P<0.0001. **(G)** Representative images of zebrafish xenograft 1-day post injection (dpi) and 4 dpi. Red indicates dyed H460 resistant cells. **(H)** NuBCP-9 suppresses growth of paclitaxel resistant H460 cells in a zebrafish xenograft model. Growth of H460 paclitaxel resistant cells in xenograft zebrafish model, pre-treated with vehicle or NuBCP-9 (10 μM) for 6 hours prior to injection into zebrafish. Paclitaxel-resistant H460 cells were pre-treated with NuBCP-9 to ensure delivery of the peptide and avoid absorption issues in zebrafish embryos. n= 34 for vehicle and n = 27 for NuBCP-9. Results are the mean±SEM of two independent experiments. Students t-test, ^*^P<0.05.

NuBCP-9 is a nine-amino acid peptide derived from Nur77 that recapitulates the effects of Nur77 localization, Bcl-2 binding and conversion of Bcl-2 function [30]. Having shown that Nur77 peptide expression induces apoptosis in paclitaxel resistant H460 cells, we next tested the effects of cell penetrating NuBCP-9. NuBCP-9 reduced growth of both parental and paclitaxel resistant H460 cells, whereas NuBCP-9/AA mutant, which does not induce apoptosis, had no effect (Figure 4A) [30]. NuBCP-9 induced apoptosis preferentially in the paclitaxel resistant H460 cells after 24 hours (Figure 4B). To confirm whether NuBCP-9 induced apoptosis was linked to conformational changes in Bcl-2 and resulting Bcl-2 functional conversion, exposure of BH3 domain was determined by Bcl-2 BH3 domain antibody [28, 30]. Paclitaxel resistant H460 cells were treated for 24 hours with 10 μM NuBCP-9 then immunostained with Bcl-2 BH3 domain antibody and analysed by flow cytometry. NuBCP-9 treatment resulted in a strong enhancement of BH3 domain fluorescence, indicating Bcl-2 conformational changes (Figure 4C). A Bcl-2 conformation independent antibody was used as a control for immunostaining both parental and resistant H460 cells treated for 24 hours with vehicle and NuBCP-9, which resulted in no shift in fluorescent peaks (Figure 4C). NuBCP-9 also reduced viability and induced apoptosis of multidrug resistant H69AR cells (Figure 4D-4E). Approximately 26% of cells were apoptotic upon treatment with 10 μM of NuBCP-9 for 36 hours (Figure 4E). We also tested effectiveness of NuBCP-9 on multidrug resistant H69AR cancer cells in 3D spheroid cultures [37]. The viability of the 3D H69AR spheroids was reduced by 60% after treatment with 10 μM NuBCP-9 for 72 hours (Figure 4F).

### NuBCP-9 prevents paclitaxel resistant lung cancer cell growth in a zebrafish xenograft model

To determine if NuBCP-9 suppress growth of paclitaxel-resistant lung cancer cells *in vivo*, we employed a zebrafish xenograft model. Paclitaxel-resistant H460 cells were fluorescently dyed and then pre-treated with peptide or vehicle for 6 hours before transplantation into the yolk sac of zebrafish embryos. Zebrafish were imaged at 1-day post injection (dpi) and 4 dpi and growth of injected cancer cells was determined. NuBCP-9 strongly suppressed paclitaxel resistant lung cancer xenograft tumor growth in zebrafish (Figures 4G-4H).

## DISCUSSION

**Figure 5:**
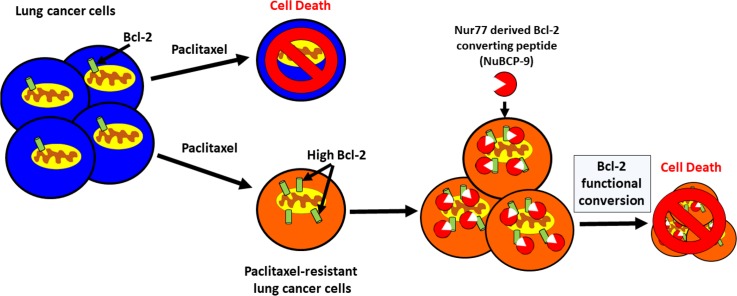
Bcl-2 functional conversion induces apoptosis in paclitaxel resistant lung cancer cells with increased expression of Bcl-2 Lung cancer cells that have acquired resistance to paclitaxel express increased levels of Bcl-2 compared to the paclitaxel sensitive cells. High Bcl-2 in the chemoresistant cells confers increased sensitivity to Nur77 derived Bcl-2 converting peptide (NuBCP-9). NuBCP-9 converts Bcl-2 from a pro-survival protein into a killer protein through a conformational change exposing its BH3 domain and induces apoptosis in the paclitaxel resistant cells.

Paclitaxel is a core component of chemotherapeutic treatment for lung cancer patients [38]. However, the clinical benefit of paclitaxel is often negated by the emergence of acquired resistance [39]. Acquired resistance can occur after several cycles of paclitaxel based chemotherapy and leads to a very poor prognosis and limited treatment options [40, 41]. In the current study, we derived paclitaxel-resistant H460 human lung cancer cells with weekly treatments for over a period of 6 weeks using concentrations within clinically acceptable safety margins of <50 nM (Figures 1A-1E) [42]. We noted that these paclitaxel resistant cancer cells were also less sensitive to doxorubicin, another commonly used chemotherapeutic drug (Figures 1B-1C). We found that expression of anti-apoptotic Bcl-2 was increased in the H460 resistant cancer cells, which may contribute towards paclitaxel resistance (Figure 2A). Multidrug resistant lung cancer H69AR cells also express high levels of Bcl-2 (Figure 2B). We further determined that the paclitaxel resistant cancer cells with elevated Bcl-2 were more susceptible to a Nur77 peptide capable of converting Bcl-2 from an anti-apoptotic protein to a pro-apoptotic protein (Figures 3A-3C). We demonstrated that NuBCP-9, a Nur77 derived peptide, reduced multidrug resistant cell viability and strongly suppressed paclitaxel resistant cancer cell growth in a zebrafish xenograft model (Figures 4D-4H). NuBCP-9 treatment exposed the Bcl-2 BH3 domain and induced apoptosis preferentially in paclitaxel resistant H460 cancer cells (Figure 4B-4C). Together, these results indicate that targeting overexpression of Bcl-2 with Bcl-2 functional converting peptides is a viable strategy to eliminate resistant cancer cells (Figure 5).

Our study demonstrates that anti-apoptotic Bcl-2 is upregulated in lung cancer cells that develop acquired resistance to standard of care chemotherapeutic paclitaxel (Figure 2A-2B). Bcl-2 was also phosphorylated at the serine 70 residue within the loop domain in the paclitaxel resistant cancer cells, which enhances Bcl-2 anti-apoptotic activity (Figure 2C) [35, 43]. Bcl-2 expression alone can confer paclitaxel resistance in H460 lung cancer cells (Figure 2G). Prior studies have implicated the Bcl-2 family of proteins in predicting response and acquired resistance to chemotherapeutic agents [33, 44, 45]. Consistently, paclitaxel resistant H460 cells with high Bcl-2 expression were cross resistant to doxorubicin (Figures 1A-1B and 2A). Interestingly H69AR cells which were derived from H69 through exposure to doxorubicin were also identified as cross resistant to paclitaxel [11]. Cross-resistance is common in patients that have become unresponsive to initial chemotherapy treatment, which limits treatment options further [46].

Our major finding in this study is that Nur77 peptide preferentially induces apoptosis in paclitaxel resistant lung cancer cells. This highlights an exciting strategy of utilizing Bcl-2 functional conversion to target chemoresistant cancers. Prior studies have shown Nur77 binds to the Bcl-2 loop domain and converts Bcl-2 from a protector to a killer protein [28, 30]. This strategy contrasts other approaches that target the survival function of Bcl-2 [28, 31, 47]. Whilst targeting Bcl-2 with inhibitors such as ABT-263 and ABT-199 has shown promise in lymphoid cancers, there has also been emergence of resistance to these therapies [48, 49]. Some potential resistance mechanisms have implicated upregulation of other anti-apoptotic proteins resulting in resistance [49, 50]. NuBCP-9, through functional conversion of Bcl-2 also inhibits other anti-apoptotic family members such as Bcl-xL [30]. This highlights the potential of the Bcl-2 functional conversion strategy to treat and prevent therapy resistant cancers. Interestingly, induction of apoptosis increased dramatically, when paclitaxel is combined with Nur77 peptide (Figure 3C). This indicates Bcl-2 functional conversion methods could be combined with existing chemotherapeutics to enhance therapeutic efficacy and sensitize resistant cancer cells to paclitaxel.

To demonstrate Bcl-2 functional conversion as a feasible approach to treat therapy resistant cancer, NuBCP-9, a Nur77 derived peptide, was tested in a zebrafish xenograft model. Cancer cells transplanted into zebrafish are exposed to a complex 3D microenvironment providing conditions more like a human cancer environment [51–54]. After treatment with NuBCP-9, paclitaxel resistant cells were unable to establish themselves and grow within the zebrafish microenvironment (Figure 4G-4H). This study highlights the role of Bcl-2 in therapy resistance and the ability to target and eliminate paclitaxel resistant cells through Bcl-2 functional conversion.

In conclusion, our results demonstrate Bcl-2 functional conversion through Nur77 peptide, NuBCP-9 is an exciting strategy to target paclitaxel and doxorubicin resistant lung cancer cells. As Bcl-2 upregulation has been implicated in resistance mechanisms to other therapeutic agents, such as cisplatin, in multiple cancer types, there is great potential to use NuBCP-9 enabled Bcl-2 functional conversion to treat an array of therapy resistant cancers [14, 33, 55, 56].

## MATERIALS AND METHODS

### Cell culture

Cell lines were obtained from ATCC (Manassas, VA, USA) and maintained according to manufacturer's instructions. The human lung cancer cell line NCI-H460, H69AR (ATCC) were cultured in RPMI medium (Corning, Manassas, VA, USA) containing 10% FBS (VWR Life Science, Radnor, PA, USA), 100 U/mL penicillin, and 100 mg/mL streptomycin (Corning). All cell lines were maintained at 5% CO_2_ and 37°C. Paclitaxel resistant H460 cells were derived by treating cells once a week over 6 weeks, initially with 1 nM paclitaxel and then increasing dose incrementally up to 100 nM. Once resistance was confirmed, paclitaxel was withdrawn from the cells. Resistance to paclitaxel was maintained without presence of paclitaxel measured up to 2 months. A parental line was maintained for a similar number of passages as the resistant line. H460 paclitaxel resistant cells were cultured in paclitaxel free media for a minimum of 3 weeks prior to all experiments.

### Generation of Bcl-2 knock out cells

Bcl-2 knockout was performed as previously reported with modifications [57]. H69AR cells were seeded at a density of 1 × 10^6^ cells per well in 6-well plates and incubated overnight. Cells were transfected the next day at 80-90% confluency with 2 μg of the indicated plasmid DNA using Lipofectamine 2000 transfection reagent (Thermo Fisher Scientific, Walktham, MA, USA). The transfected cells were selected by culturing in medium containing 2.5 μg/mL puromycin. Puromycin was removed one week prior to experiments. pLentiCRISPR v2 control vector was a gift from Feng Zhang (Addgene plasmid # 52961). The Bcl-2 targeting CRISPR-Cas9 vectors used in this study were obtained from GenScript (Piscataway, NJ, USA). The guide RNA sequence used in this study were: Bcl-2-1, 5′-ACCTGACGCCCTTCACCGCG-3′.

### Chemicals and peptides

Cycloheximide, paclitaxel and doxorubicin was purchased from Sigma Aldrich (St Louis, MO, USA). DMSO was purchased from VWR Life Sciences (Radnor, PA, USA). Cell penetrating NuBCP-9, NuBCP-9/AA was purchased from Lifetein (Lifetein, Hillsborough, NJ, USA) [30].

### 3D spheroid cultures

10,000 cells per well were plated into a non-adherent round bottom 96 well plate as per manufacturer protocol (Corning Cat. No. 4520). Cells formed spheroids for 48-72 hours prior to treatment.

### Viability assay

Cells of interest were plated at 2000 cells per well in 96-well black tissue culture plate and allowed to adhere to the plate overnight. Viability assays were performed using 10% serum medium unless otherwise stated. Drugs were diluted in supplemented tissue culture medium and added at increasing concentrations, with DMSO as a vehicle control. Cells were then incubated for either 48 or 72 hours in presence of the compound. Titer Glo (G7570, Promega, Madison, WI) was added to the wells at the assay end point according to manufacturer's protocol. Luminescence was measured using Tropix TR717 Microplate luminometer. Percentage of viable cells is relative to vehicle (100%).

### Western blotting

Analysis of protein abundance was performed by Western blot according to standard techniques. Briefly cell lysates were collected using RIPA buffer with protease inhibitor, and then quantified using BCA assay. Some cell lysates were collected using 2X Laemmli buffer directly. Samples were boiled for 5 min and ran on SDS PAGE 12% and transferred to PVDF membranes by semi-dry transfer. Blots were probed using following antibodies, Bcl-2 (sc-509, Santa Cruz Biotechnology, Dallas, TX, USA), GAPDH (sc-365062, SantaCruz), Mcl-1 (PA5-27597, Invitrogen, Carlsbad, CA, USA), Bcl-xL (AHO0222, Biosource, Waltham, MA, USA), Cleaved Caspase 3 Asp175 ( 9661, Cell Signaling, Danvas, MA, USA). Chemiluminescence signal was developed using horse radish peroxidase conjugated secondary antibodies (SouthernBiotech, USA) and SuperSignal West Pico reagent (Thermo Fisher Scientific, Waltham, MA, USA). Images were captured using a G:BOX imaging system and GeneSys software version 1.5.9 (Syngene, Cambridge, UK).

### Real time-quantitative PCR

Total RNA was prepared using total RNA kit (Omega BioTek, Norcross, GA). The first strand cDNA was synthesized using Transcriptor kit (Roche, Indianapolis, IN). Real-time qPCR was done using FastStart Universal SYBR Green master mix (Roche) in 7500 Fast PCR system (Applied Biosystems, Foster City, CA, USA) according to the manufacturer's protocol. The human primer sequences used in this study were as follows:

GAPDH forward, 5′-ACCTTTGACGCTGGGG CTGG-3′;

GAPDH reverse, 5′-CTCTCTTCCTCTTGTGCTCTTGCTGG-3′;

BCL-2 forward, 5′-GATCCTCGAGATGGCGCACGCTGGGAGAAC-3′;

BCL-2 reverse, 5′-GATCGGATCCTCATGGCTGAGCGCAG-3′;

MCL-1 forward, 5′-TGCTTCGGAAACTGGA CATCA-3′;

MCL-1 reverse, 5′-TAGCCACAAAGGCACC AAAAG-3′;

BCL-XL forward, 5′-GAGCTGGTGGTTGACTTT CTC-3′;

BCL-XL reverse, 5′-TCCATCTCCGATTCAGT CCCT-3′.

### Transfection

H460 cells were transfected with GFP-Nur77 478-504 fragment or GFP alone [30]. Transfections were performed using Lonza (Lonza, Basel, Switzerland) 4D nucleofector according to manufacturer's protocol with SE Cell Line 4D Nucleofector x Kit (cat: V4XC-1032) and program EO-100. H69AR cells were transfected with same plasmid using lipofectamine 2000 (Thermo Fisher Scientific, Waltham, MA, USA) according to manufacturer's protocol. Lipofectamine 2000 was used according to manufacturer's protocol for expression of Bcl-2 or control pcDNA plasmid in H460 cells.

### Annexin V staining for apoptosis using flow cytometry

Cells were seeded into 6 well tissue culture plates to give approximately 50% confluence and allowed to attach overnight. The cells were then treated for a period of time indicated in figure legend with the appropriate compound at various concentrations. An annexin V-conjugate PerCP-eFluor 710 apoptosis detection kit was used as described by the manufacturer's protocol (88-8008, eBioscience, Waltham, MA, USA). Harvesting of cells included collection of floating and attached cells following trypsinization. Data were acquired using an CytoFLEX S flow cytometer (Beckman Coulter, Brea, CA) and 10,000 events on the PC5.5 channel were analyzed using CytExpert software (Beckman Coulter). For Nur77 GFP tagged annexin V analysis, GFP positive population were selected and then annexin V + population was determined. For each sample, 10,000 GFP+ events were collected. annexin V positive cell population was considered as the apoptotic population, and the percent of annexin V positive cell population was used to determine the extent of apoptosis.

### Flow cytometry viability assay

Cells were seeded at 10^6^ per well in a 6 well plate and allowed to adhere overnight. The following day cells were treated with vehicle or indicated compound for 48 hours. Supernatant and attached cells were removed from plate using trypsin and washed 3 times in cold PBS. Cells were then stained using eBioscience Fixable Viability dye eFluor 660 (Cat#65-0864) (Invitrogen, Carslbad, CA) for 30 minutes on ice. Cells were then washed three times followed by fixation with 3.7% paraformaldehyde (PFA) for 10 minutes at room temperature. Fixed cells were then washed with PBS and permeabilized using Triton x-100 0.1% in PBS for 10 minutes, this was followed by three PBS washes. Cells were then resuspended in Block solution (0.1%BSA in PBS) for 1 hour at room temperature. Primary antibody Bcl-2 Fluorescein isothiocyanate (FITC) conjugate (cat# A15764) (Invitrogen, Carslbad, CA) was added at 1:100 dilution for 1 hour at room temperature followed by PBS washes. Samples were analysed using flow cytometry, gates defined Bcl-2 high population and Bcl-2 low population using FITC fluorescent channel. For Bcl-2 low and high cells, percentage of cell death was determined by fixable viability dye positivity.

### Bcl-2 BH3 conformation change assay

Cells were seeded at 10^6^ per well in a 6 well plate and allowed to adhere overnight. The following day cells were treated with vehicle or indicated compound for 24 hours. Supernatant and attached cells were removed from plate using trypsin and washed 3 times in cold PBS. Cells were fixed with 3.7% PFA for 10 minutes at room temperature. Fixed cells were then washed with PBS and permeabilized using Triton x-100 0.1% in PBS for 10 minutes, this was followed by three PBS washes. Cells were then resuspended in Block solution (0.1%BSA in PBS) for 1 hour at room temperature. Primary antibody Bcl-2 BH3 domain specific (cat# AP1303a) (Abgent, San Diego, CA) was added at a 1:30 dilution overnight at 4°C and then washed three times using cold PBS. For control experiment primary antibody Bcl-2(100) (cat# MA5-11757) (Thermo Fisher Sci, Waltham, MA, USA) was added at 1:50 dilution overnight at 4°C and then washed three times using cold PBS. Secondary antibody FITC conjugate was added at 1:100 dilution in block solution for 1 hour at room temperature. Samples were analysed using flow cytometry, shift of peak to the right in FITC channel determines extent of Bcl-2 BH3 exposure.

### Xenograft study

Zebrafish (*Danio rerio*) were housed at the Sinnhuber Aquatic Research Laboratory at Oregon State University in accordance with Institutional Animal Care and Use Committee protocols. Adult 5D Tropical zebrafish were maintained under standard laboratory conditions of 28±1°C on a 14hr light/10 hr dark photoperiod in fish water consisting of reverse osmosis water supplemented with a commercially available salt solution (0.6%, Instant Ocean, UnitedPet Group, Inc., Blacksburg, VA, USA). Collected eggs were staged according to Kimmel et al [58]. At 24 hours post fertilization (hpf), zebrafish embryos were maintained in E3 media with phenylthiourea (0.003%, Sigma, USA).

Xenograft transplantation protocols were adapted from [51]. Briefly, H460 cells were labeled with a CM-DiI dye (Thermo Fisher Sci.) according to the manufacturer's protocol and suspended to a concentration of 2×10^7^ cells/mL. Cell suspension was loaded into a borosilicate glass needle pulled from a pipette by a micropipette puller (Sutter Instrument, Novato, CA). Approximately 200 H460 cells were transplanted into the yolk of 48 hpf embryos by air-driven micro-pressure injector. After transplantation, embryos recovered overnight at 33°C without light.

For imaging, zebrafish xenografts were anesthetized by emersion in 0.2 mg/mL Tricaine E3 media and imbedded in 0.8% (w/v) low melting point agarose on a glass bottom 96-well plate. A Zeiss LSM 780 confocal microscope with a 10x objective was used to capture fluorescent cells at 1 and 4-day post injection (dpi). Images were captured as z-stacks with wide-field settings. H460 cancer growth was analyzed using Fiji (Fiji is Just ImageJ) software [59]. Images were processed by making a maximum projection image of the z-stack and using a median filter. Cancer area was calculated by creating a binary mask from thresholds with the Otsu algorithm and calculating the total area of the resulting segmented objects. Increases in total area from 1 to 4dpi were considered cancer growth.

### Data analysis

Viability data were analyzed by one-way ANOVA with multiple comparison post-test using Prism software (Version 7.03, Graphpad Software, La Jolla, CA). P values less than 0.05 were considered statistically significant.
